# Supplementary material for: Effect of Contact Area and Shape of Anode Current Collectors on Bacterial Community Structure in Microbial Fuel Cells
Source: Molecules. 2022 Mar 30;27(7):2245. doi: 10.3390/molecules27072245 (PMC9000358; doi:10.3390/molecules27072245)
Supplement: Supplementary file 1 [file molecules-27-02245-s001.zip › molecules-1622666-supplementary.pdf]

Supplementary Materials

Effect of Contact Area and Shape of Anode Current Collectors on Bacterial Community Structure in Microbial Fuel Cells

Agathe Paitier [1,2], Naoufel Haddour [1,*], Chantal Gondran [3] and Timothy M. Vogel [2]

[1] Laboratoire Ampère, Université de Lyon, Ecole Centrale de Lyon, CNRS, UMR 5005, 36 Avenue Guy de Collongue, 69134 Ecully, France; naoufel.haddour@ec-lyon.fr
[2] Environmental Microbial Genomics, Laboratoire Ampère, Université de Lyon, Université Claude Bernard Lyon 1, CNRS, UMR 5005, 43 Boulevard du 11 Novembre 1918, CEDEX, 69616 Villeurbanne, France; vogel@univ-lyon1.fr
[3] DCM, Université Grenoble Alpes, CNRS, Grenoble F-38000, France; chantal.gondran@univ-grenoble-alpes.fr

* Correspondence: naoufel.haddour@ec-lyon.fr; (N.H.); Tel. : +33-4-72-18-61-12 (N.H)

Figure S1. Maximal power output for each current collector configuration.

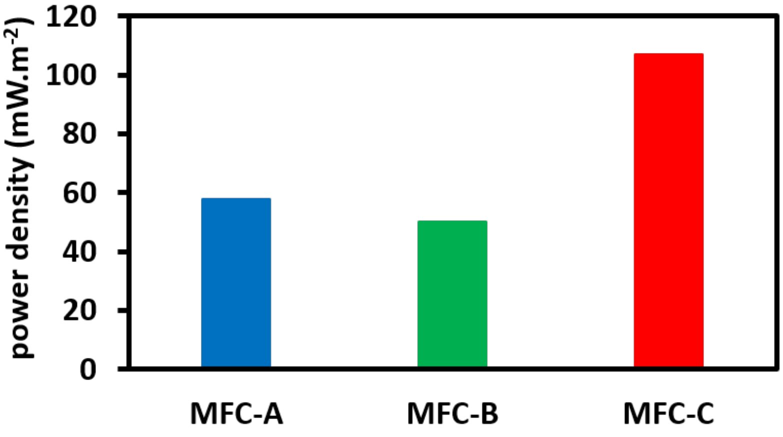

Table S1. Composition of synthetic wastewater for 500mg COD/L.

Compounds dissolved in tap water	Mass for 1L of solution (mg)
tryptone	800
yeast extract	550
urea (CH_4N_2O)	150
anhydrous dipotassium hydrogen phosphate (K_2HPO_4)	140
sodium chloride (NaCl)	35
calcium chloride dihydrate ($CaCl_2.2H_2O$)	20
magnesium sulphate heptahydrate ($MgSO_4.7H_2O$)	10

Table S2. Primer sequences for qPCR assay of "all bacteria" and for *Geobacter* sp. and for sequencing of the variable V3 and V4 regions of the 16S rRNA gene.

Assay	Names and sequences (5'-3')	Product size (bp)	Annealing temperature (°C)	Reference
"all bacteria"	**Eub338F** ; ACT CCT ACG GGA GGC AGC AG<br>**Eub518R** ; ATT ACC GCG GCT GCT GG	200	53	Fierer *et al.* [1]
Geobacter sp.	**Geo561F** ; GCG TGT AGG CGG TTT CTT AA<br>**Geo825R** ; TAC CCG CRA CAC CTA GTT CT	265	59	Stults *et al.* [2]
Primers for 16S V3 and V4 regions	**Fwd** ; TCG TCG GCA GCG TCA GAT GTG TAT AAG AGA CAG CCT ACG GGN GGC WGC AG<br>**Rev** ; GTC TCG TGG GCT CGG AGA TGT GTA TAA GAG ACA GGA CTA CHV GGG TAT CTA ATC C	460	55	Illumina's instructions [3]
